# Supplementary material for: The Prevalence and the Impact of Frailty in Hepato-Biliary Pancreatic Cancers: A Systematic Review and Meta-Analysis
Source: J Clin Med. 2022 Feb 20;11(4):1116. doi: 10.3390/jcm11041116 (PMC8878959; doi:10.3390/jcm11041116)
Supplement: Supplementary file 1 [file jcm-11-01116-s001.zip › Supplementary Data_fig_tabR.pdf]

Supplementary Data

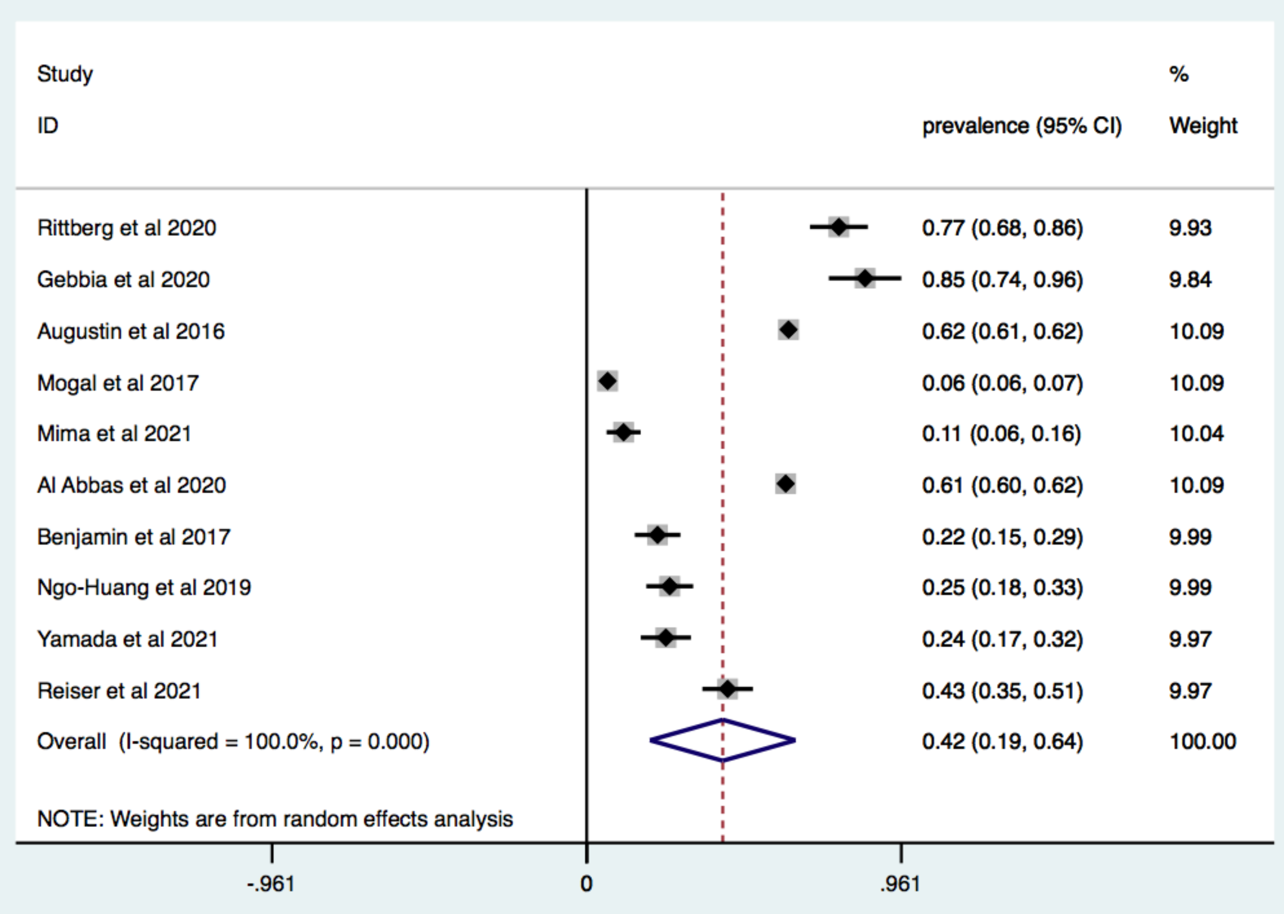

Figure S1. Overall prevalence of frailty in patients with pancreas cancer. Forest plot of cumulative prevalence of frailty in patients with pancreas cancer. Squares are study-specific prevalence. Diamond is the pooled prevalence. Horizontal lines represent 95% Confidence Interval (CI).

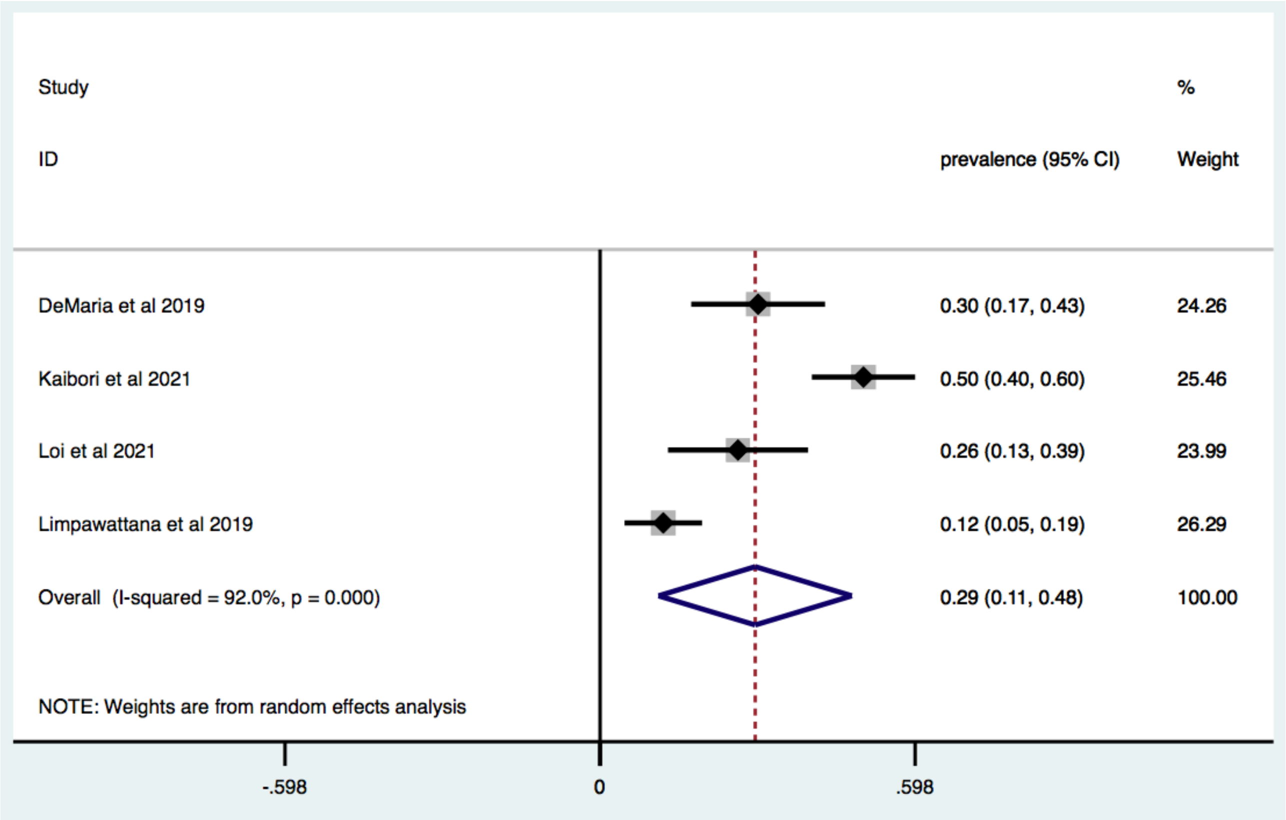

Figure S2. Overall prevalence of frailty in patients with liver cancer. Forest plot of cumulative prevalence of frailty in patients with liver cancer. Squares are study-specific prevalence. Diamond is the pooled prevalence. Horizontal lines represent 95% Confidence Interval (CI).

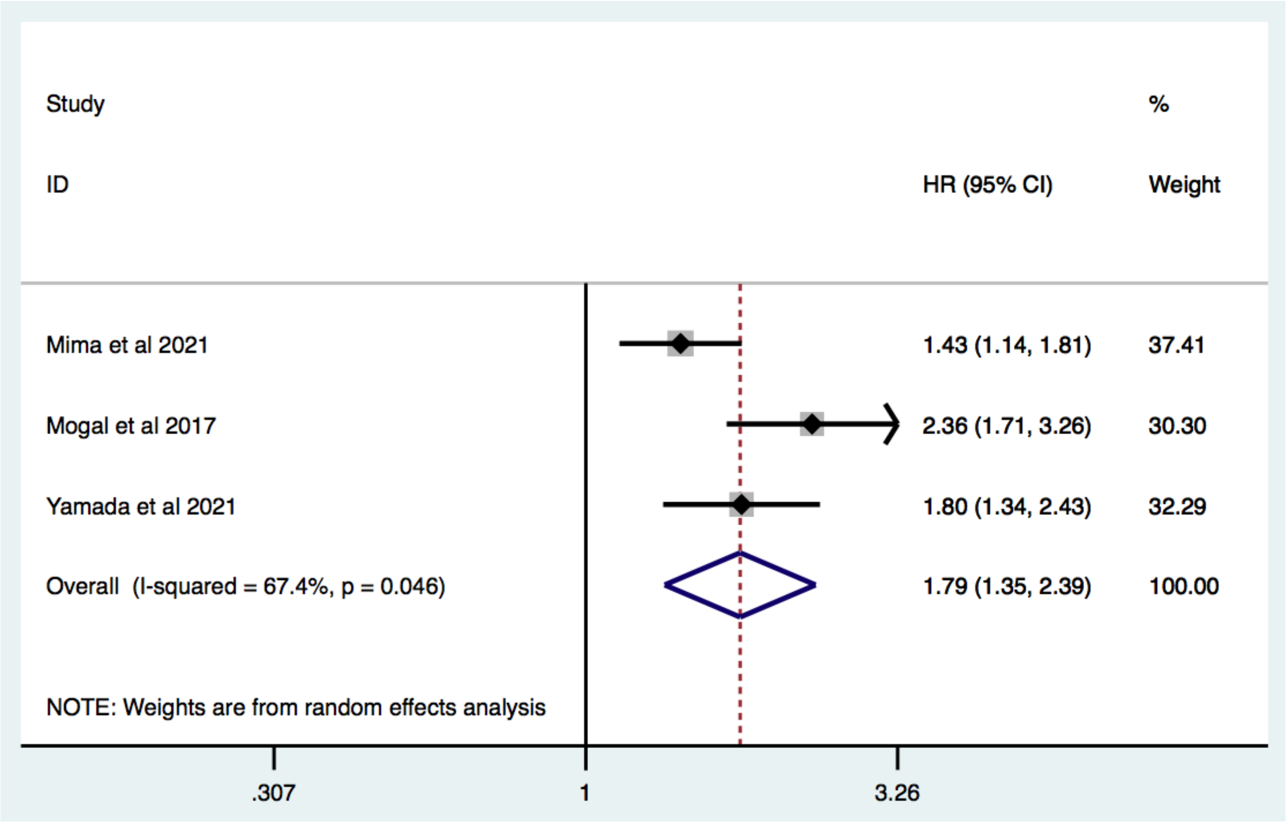

Figure S3: Mortality risk in frail vs non frail patients with HPB cancer where surgery treatment was performed. Squares are study-specific Relative Risk (RR). Diamond is the estimated overall RR. Horizontal lines represent 95% Confidence Interval (CI).

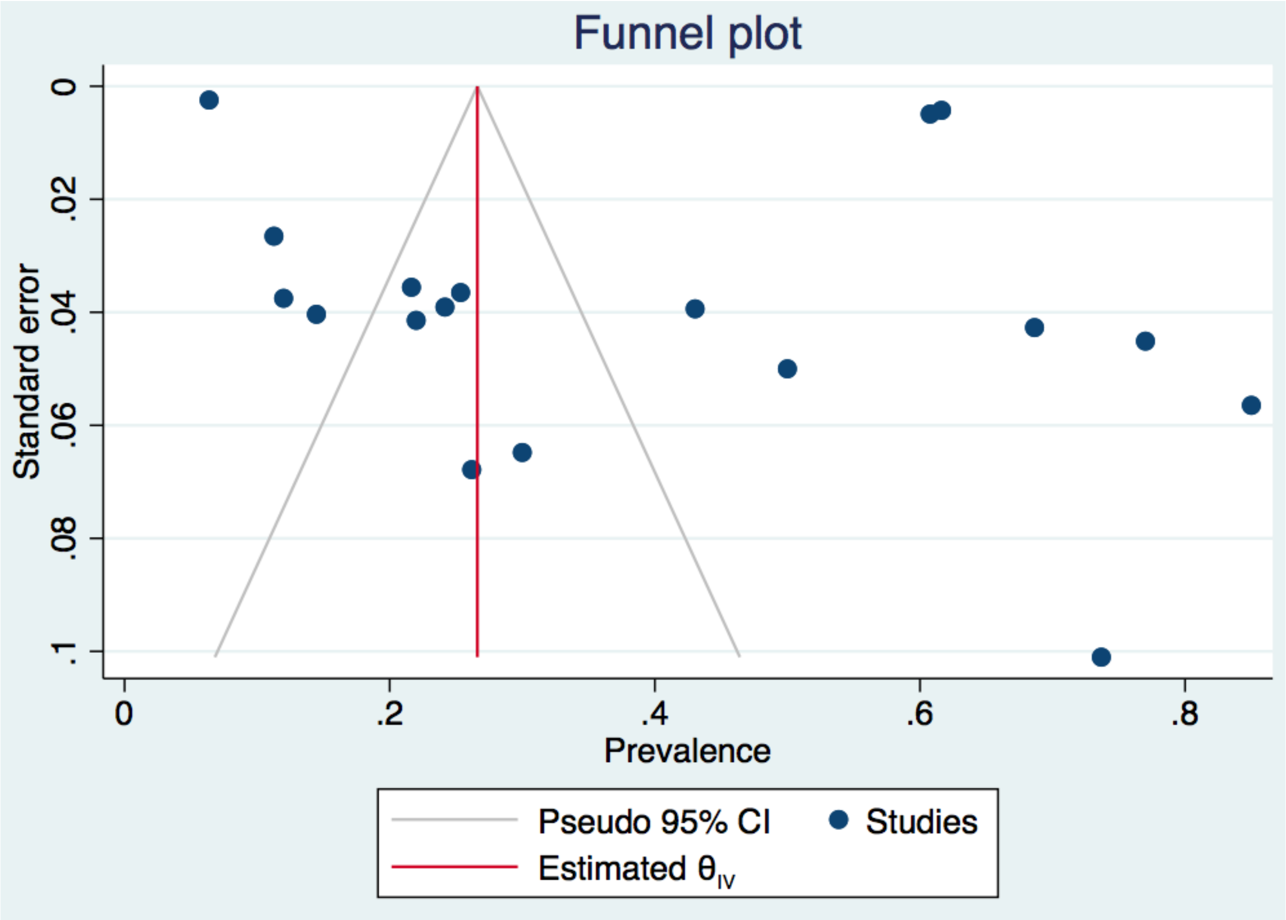

Figure S4: funnel plot of all prevalence studies

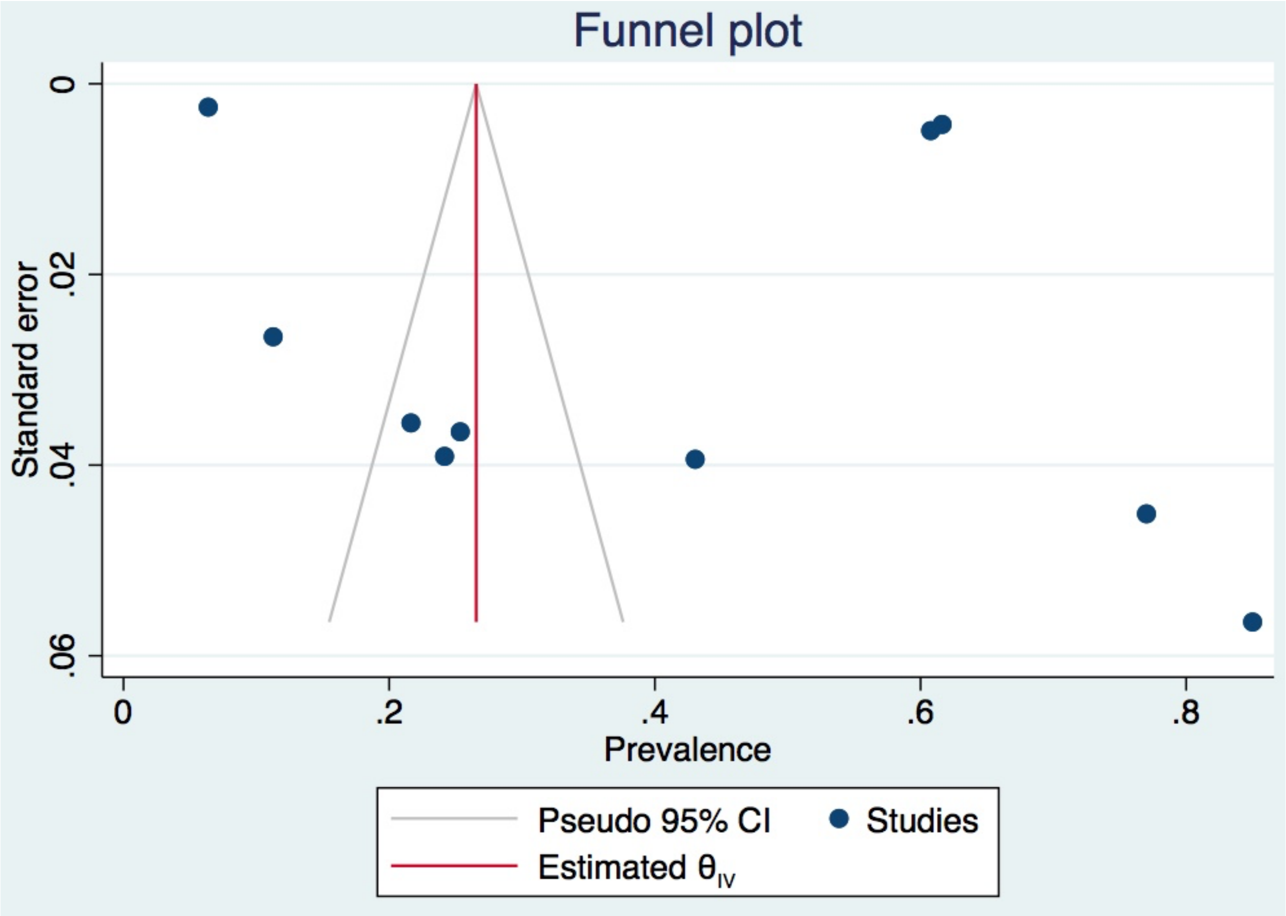

Figure S5: funnel plot of prevalence studies including only pancreas cancer

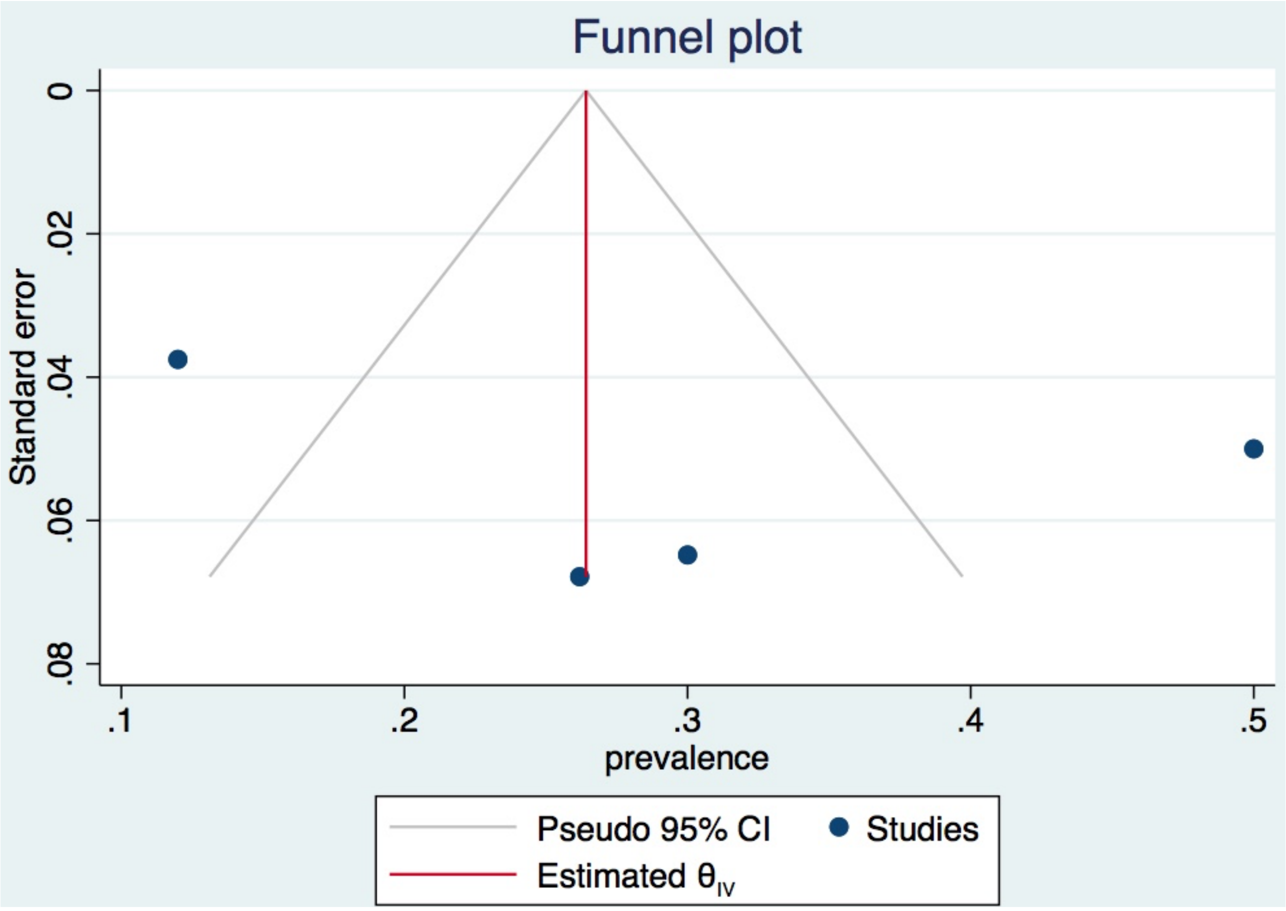

Figure S6: funnel plot of prevalence studies including only liver cancers

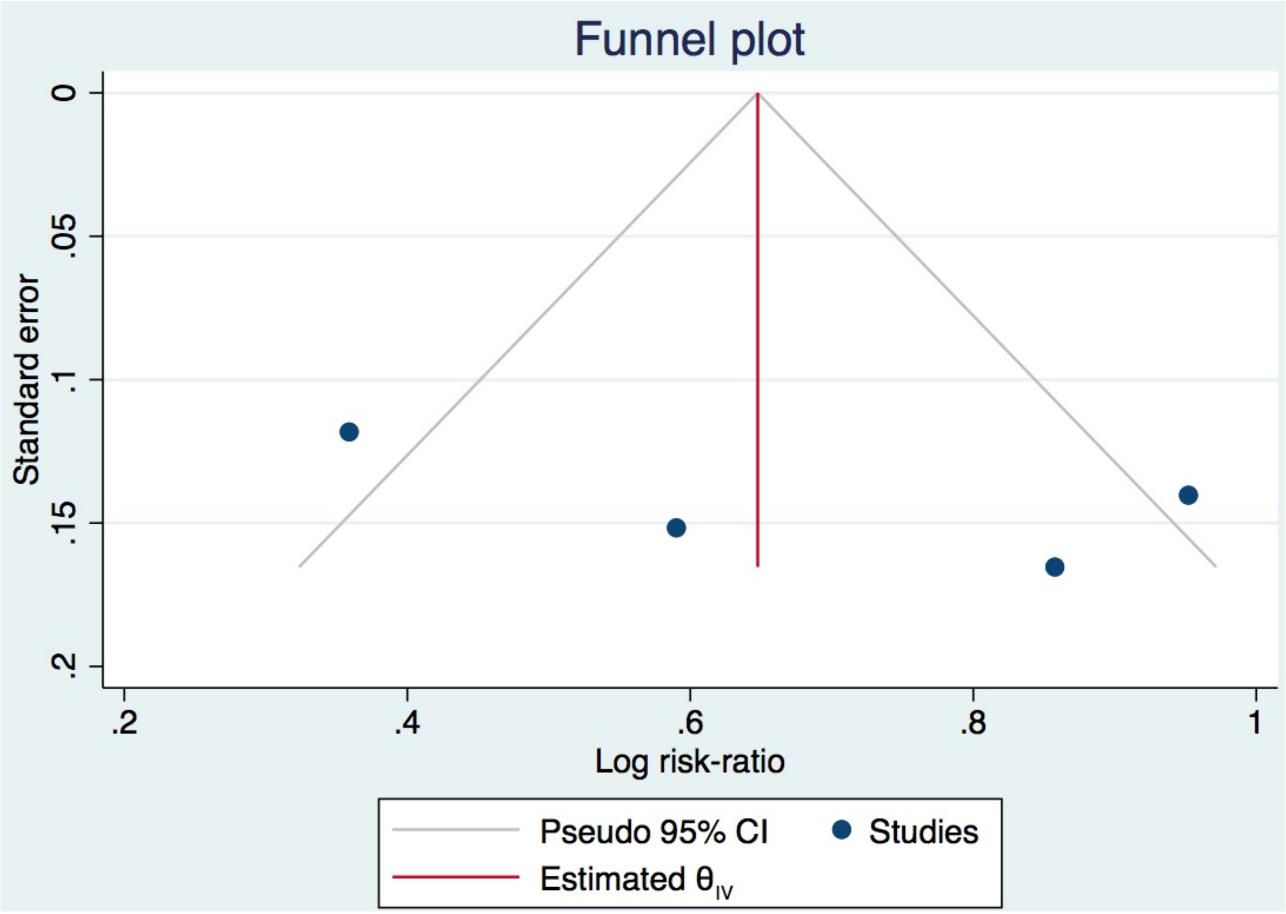

Figure S7: funnel plot of mortality risk

Table S1 . Study Quality.

Study	Case definition adequate	Representativeness of the cases	Selection of controls	Definition of controls	Comparability based on design or analysis	Ascertainment of exposure	Same method of ascertainment for case and controls	Non response rate	Total Score
Al Abass et al 2020	★	★	★	★	★★	-	★	-	7
Augustin et al 2016	★	★	★	★	★★		★	-	7
Baimas-George et al 2021	★	★	★	★	★★		★	-	7
Benjamin et al 2017	★	★	★	★	-	-	★	-	5
Dale et al 2014	★	★	★	★	-		★		5
DeMaria et al 2019	★	★	★	★	★	★	★	-	7
Gebbia et al 2020	★	★	★	★	★	★	-	-	6
Kaibori et al 2021	★	★	★	★	★	★	★	-	7
Limpawattana et al 2019	★	★	★	★	★	-	-	-	5
Loi et al 2021	★	★	★	★	★		-	-	5
Mima et al 2021	★	★	★	★	★	-	★	-	6
Mogal et al 2017	★	★	★	★	★	-	-	-	5
Ngo-Huang et al 2019	★	★	★	★	★	-	★	-	6
Reiser et al 2021	★	★	★	★	★★	-	★	-	7
Rittberg et al 2020	★	★	★	★	★	-	★	-	6
van Wijk et al 2021	★	★	★	★	★★	-	★	-	7
Yamada et al 2021	★	★	★	★	★★	★	★	-	8
Paolini et al 2021	★	★	★	★	★	★	★	-	7
